# Biological Risk Assessment of Three Dental Composite Materials following Gas Plasma Exposure

**DOI:** 10.3390/molecules27144519

**Published:** 2022-07-15

**Authors:** Sander Bekeschus, Lea Miebach, Jonas Pommerening, Ramona Clemen, Katharina Witzke

**Affiliations:** 1 ZIK *plasmatis*, Leibniz Institute for Plasma Science and Technology (INP), Felix-Hausdorff-Str. 2, 17489 Greifswald, Germany; lea.miebach@inp-greifswald.de (L.M.); jonas.pommerening@inp-greifswald.de (J.P.); ramona.clemen@inp-greifswald.de (R.C.); 2 Department of General, Vascular, Thoracic, and Visceral Surgery, Greifswald University Medical Center, Ferdinand-Sauerbruch-Str., 17475 Greifswald, Germany; 3 Department of Oral, Maxillofacial, and Plastic Surgery, Greifswald University Medical Center, Ferdinand-Sauerbruch-Str., 17475 Greifswald, Germany; katharina.witzke@med.uni-greifswald.de

**Keywords:** atmospheric pressure argon plasma jet, plasma medicine, reactive oxygen species, resin

## Abstract

Gas plasma is an approved technology that generates a plethora of reactive oxygen species, which are actively applied for chronic wound healing. Its particular antimicrobial action has spurred interest in other medical fields, such as periodontitis in dentistry. Recent work has indicated the possibility of performing gas plasma-mediated biofilm removal on teeth. Teeth frequently contain restoration materials for filling cavities, e.g., resin-based composites. However, it is unknown if such materials are altered upon gas plasma exposure. To this end, we generated a new in-house workflow for three commonly used resin-based composites following gas plasma treatment and incubated the material with human HaCaT keratinocytes in vitro. Cytotoxicity was investigated by metabolic activity analysis, flow cytometry, and quantitative high-content fluorescence imaging. The inflammatory consequences were assessed using quantitative analysis of 13 different chemokines and cytokines in the culture supernatants. Hydrogen peroxide served as the control condition. A modest but significant cytotoxic effect was observed in the metabolic activity and viability after plasma treatment for all three composites. This was only partially treatment time-dependent and the composites alone affected the cells to some extent, as evident by differential secretion profiles of VEGF, for example. Gas plasma composite modification markedly elevated the secretion of IL6, IL8, IL18, and CCL2, with the latter showing the highest correlation with treatment time (Pearson’s r > 0.95). Cell culture media incubated with gas plasma-treated composite chips and added to cells thereafter could not replicate the effects, pointing to the potential that surface modifications elicited the findings. In conclusion, our data suggest that gas plasma treatment modifies composite material surfaces to a certain extent, leading to measurable but overall modest biological effects.

## 1. Introduction

Gas plasma is a partially ionized gas capable of generating various reactive oxygen and nitrogen species (ROS) when in contact with ambient air oxygen and nitrogen [1]. A leap in innovation in plasma technology development initiated the investigation of gas plasma sources operated at atmospheric pressure, exhibiting temperatures of only approximately body temperature [2]. Approximately two decades ago, such tissue-compatible gas plasma devices were found to exhibit potent antimicrobial effects and were soon after suggested for biomedical applications [3,4]. Excessive bacterial and fungal growth is an issue, especially in chronic wounds and ulcers. Subsequently, healing chronic wounds and ulcers using gas plasma technology was investigated [5]. Approximately one decade ago, the first medical plasma devices were approved for clinical application based on experience in patient studies [6].

Meanwhile, gas plasma applications have also spurred interest in other medical fields, such as dentistry, where this novel technology may address several clinical challenges. Gas plasma applications in the oral cavity include the treatment of implant-associated infection [7], oral candidiasis [8], and periodontitis [9], with the latter having been already successfully demonstrated in a randomized clinical trial [10]. In addition, improved osteoblast adhesion and differentiation have been investigated following gas plasma exposure of cells or respective materials [11]. In general, gas plasma in the oral cavity either directly targets tooth structures or is operated in close vicinity to the teeth, such as the gum. Teeth frequently contain restoration materials for filling cavities, e.g., resin-based composites; however, it is unknown if the alteration of such structures upon gas plasma exposure affects nearby cells. For instance, they could be rendered more cytotoxic or change the inflammatory secretion profiles of cells.

To this end, we tested three commonly used resin-based composite filling materials (ArabeskTOP, ArabeskFLOW, and GrandioSO) following gas plasma treatment to perform a biological risk assessment. To provide reproducible treatment, we generated a novel in-house workflow for composite chip manufacturing and automated gas plasma exposure using a computer-controlled *xyz*-stage. Subsequently, human HaCaT keratinocytes were cultured on the gas plasma-treated side of the composite chips, and their viability and inflammatory secretion profiles were assessed. A keratinocyte cell line was used as keratinocytes are part of the human oral mucosa, which can be in contact with dental material. The immortalized but non-malignant cell line has been previously suggested as a substitute for oral keratinocytes [12] and has been utilized in previous safety studies in plasma medicine [13]. Our data revealed several cellular perturbations as a consequence of gas plasma exposure.

## 2. Results

### 2.1. Composite Manufacturing, Gas Plasma Treatment, and Cell Culture Workflow Setup

**Figure 1:**
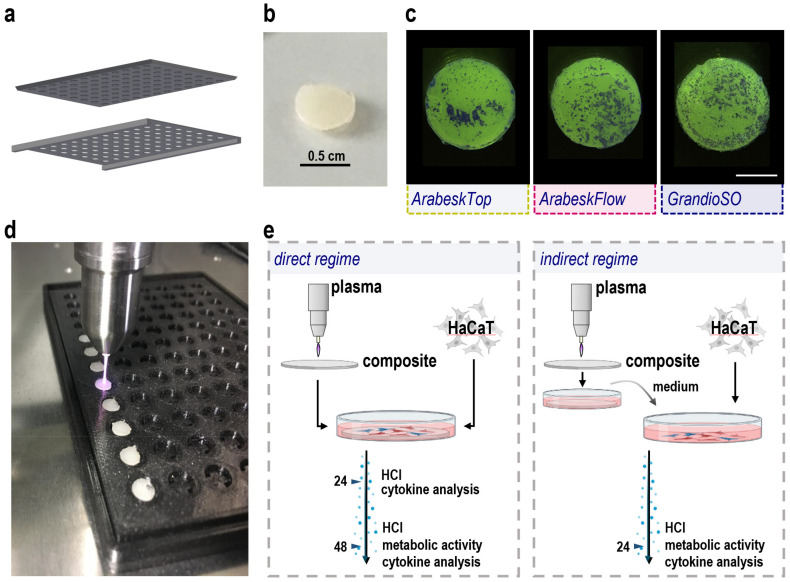
**Composite chip manufacturing and treatment.** (**a**) rapid prototyping of 3D-printed 96-well schemes for insertion of a defined amount of dental composite material prior to material hardening using a standard dental UV lamp for a predetermined time; (**b**) image of a standardized composite chip generated with the workflow; (**c**) stereo-microscopy of composite chips incubated with human HaCaT keratinocytes for 48 h, fixation of the chips and cells, and staining using crystal violet, providing evidence of cellular material adherent to the chips; (**d**) *xyz*-stage automated gas plasma treatment of the center of each chip added to a customized 3D-printed vented 96-well holder for the treatment times 30 s, 60 s, and 120 s; (**e**) experimental schemes of (**e**) direct and indirect gas plasma treatment workflows and (24 h, 48 h) incubation times (HCI = high content imaging) (designed using biorender.com).

**Figure 2:**
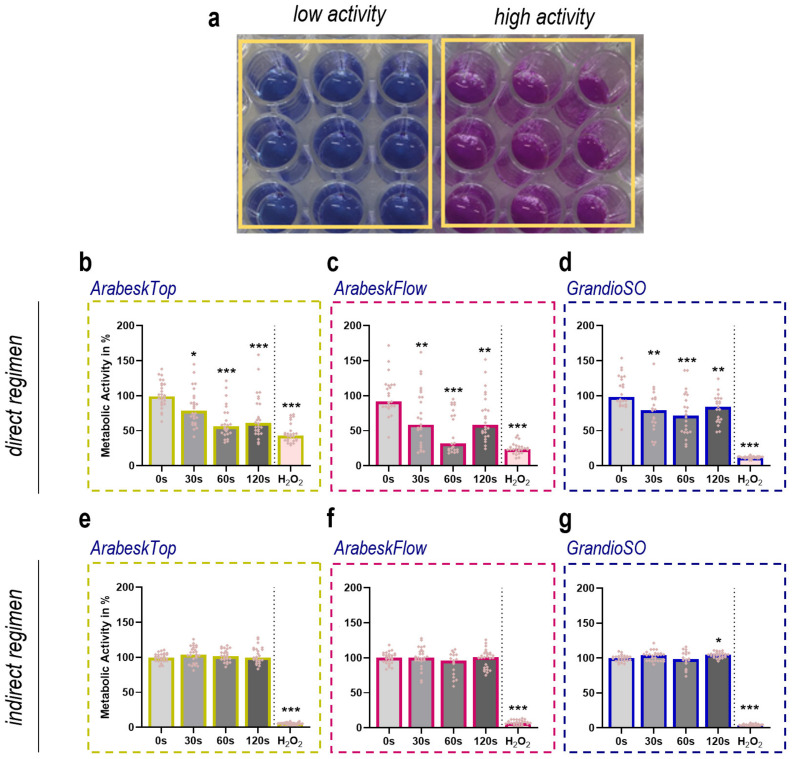
**Human HaCaT keratinocyte metabolic activity.** (**a**) representative wells of the resazurin-based assay as a measure of reduction equivalents present in cell cultures; (**b**–**d**) resorufin fluorescence of human HaCaT keratinocytes cultured in the presence of untreated (0 s) or gas plasma-treated ArabeskTop (**b**), ArabeskFlow (**c**), and GrandioSO (**d**) in cell culture media in 96-well plates for 24 h (direct regimen); (**e**–**g**) resorufin fluorescence of human HaCaT keratinocytes cultured in the absence of composite chips but in the presence of cell culture medium incubated for 24 h with untreated (0 s) or gas plasma-treated ArabeskTop (**e**), ArabeskFlow (**f**), and GrandioSO (**g**) composite chips (indirect regimen). Hydrogen peroxide (H_2_O_2_)-treated composite chips served as a positive control. Data are normalized to untreated (0 s) conditions and are mean of six independent experiments with three technical replicates each. Statistical analysis was performed using one-way analysis of variances with * = *p* < 0.05, ** = *p* < 0.01, and *** = *p* < 0.001 and Dunnett’s post-hoc test for untreated control conditions (0 s).

To investigate the biological consequences of gas plasma-treated composite material in a reproducible in vitro setting, several challenges needed to be addressed. Cell cultures are often performed in 96-well plates with modified bottoms to allow adherent cells to adhere. To use this existing system, we generated composite chips with approximately the same area and diameter as the wells of the 96-well plate by filling the liquid composites into a customized 3D-printed template (Figure 1a). Simultaneously, the goal was to achieve a small chip height to save composite material and retain maximal translucency of the material for imaging experiments. To this end, a glass plate was added to the well plate, ensuring a similar height across all composite chips generated before these were hardened using a standard dentistry UV lamp (Figure 2b). In pilot experiments, all three composite types (Table 1) were added to 96-well plates, and human HaCaT keratinocyte cell suspensions were given to the composites. Two days later, the cells were washed, fixed, and crystal violet stained. Cellular presence and growth were found on all three composite types (Figure 1c). Next, a reproducible and standardized gas plasma exposure workflow was created. The aim was to perform gas plasma exposure centrally on each chip at precisely the same height (the distance from the argon plasma jet to the composite surface) and for exact treatment times. Therefore, a customized 3D-printed template was generated to hold the composite chips in place during the treatment. Because the chips were lightweight and the argon gas pressure of the gas plasma jet can be substantial, especially when directly connected (conductive) to the target [14], some tended to be flip over during the treatment or the movement of the jet from one chip to another. To solve this, venting slits were added to the composite chip cavities (Figure 1d). Highly reproducible gas plasma exposure was achieved by connecting the plasma jet to a motorized and computer-controlled *xyz*-stage. This setup allowed the addressing of two questions: Firstly, how are human cells affected by gas plasma treatment in comparison to untreated dental composites when cultured directly on the surface (Figure 1e, left image)? Secondly, is the gas plasma exposure ablating any surface dental composite material which can diffuse into liquids, and would such liquids containing ablated material affect cellular properties (Figure 1e, right image)? Several cell biological assays were performed to address these questions.

### 2.2. Cytotoxicity Analysis of Three Types of Gas Plasma-Treated Dental Composites

**Figure 3:**
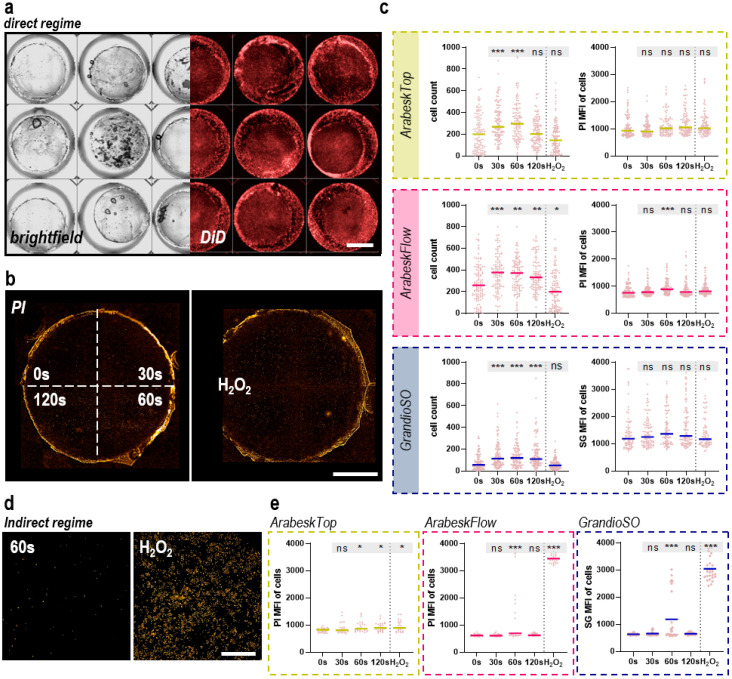
**Quantitative fluorescence imaging.** (**a**) brightfield (left) and fluorescently labeled (DiD) human HaCaT keratinocytes grown on composite chips cultured in a tissue culture-treated 96-well plate with each well image being digitally stitched from nine individual fields of view with three channels (brightfield, DiD, PI) each; (**b**) representative PI imaged digitally aligned side-by-side for all gas plasma treatment conditions (in the experiments, each composite chip was exposed to a single gas plasma treatment time) of composite chips and the positive control H_2_O_2_; (**c**) quantitative algorithm-driven image analysis of the number of cells (left) and mean fluorescence intensity (MFI) of PI per cell and well (right) across the three composite chip types investigated in this study of the direct treatment and culture regimen; (**d**) representative PI images of a 60 s gas plasma-treated (left) and H_2_O_2_-treated (right) composite chips; (**e**) quantitative algorithm-driven image analysis of the number of cells (left) and mean PI intensity per cell and well (right) across the three composite chip types investigated in this study of the indirect treatment and culture regimen. Data are mean of four independent experiments with three technical replicates and several fields of view each. Statistical analysis was performed using one-way analysis of variances with * = *p* < 0.05, ** = *p* < 0.01, and *** = *p* < 0.001 and the Dunnett post-hoc test for untreated control conditions (0 s).

Three dental composites (ArabeskTop, AT; ArabeskFlow, AF; GrandioSO, GS) were gas plasma treated and cultured in the presence of human HaCaT keratinocytes. Twenty-four hours later, their metabolic activity was analyzed with an assay measuring the total reduction equivalents within a cell culture vessel (Figure 2a). There was a gas plasma treatment time-dependent decline in metabolic activity for AT (Figure 2b), albeit that the 120 s conditions seemed somewhat less toxic as compared to the 60 s conditions. The same phenomena could be observed for AF (Figure 2c), while it was less pronounced when in GS (Figure 2d). Hydrogen peroxide (H_2_O_2_) served as a positive control in all instances and H_2_O_2-_treated composite chips reduced metabolic activity as expected. Altogether, gas plasma exposure of all three composite types and direct culture of human HaCaT keratinocytes on the chips’ surface caused significant but overall moderate toxicity in these cells. Flow cytometry investigations validated these results (data not shown). In contrast, culturing HaCaT keratinocytes in a cell culture medium incubated with untreated or gas plasma-treated composite chips for 24 h did not replicate the modest cytotoxicity observed with direct culturing for either of the types tested (Figure 2e–g). To profile both conditions to a greater degree, we took advantage of the small height of the composite chips, allowing for inverted imaging of fluorescently labeled cells on the top side of the dental composite chips. The fluorescence allowed for the identification of the cells (Figure 3a), while the addition of the dead-cell fluorescence dye propidium iodide (PI) marks terminally dead cells (Figure 3b). Each chip in the well plate was imaged at several heights (z-stacks) to compensate for subtle micrometer-range yet unavoidable differences in absolute heights for imaging. Quantification of cells using AT revealed significantly higher cell numbers for the short and intermediate gas plasma treatment times, while viability was not significantly affected (Figure 3c). Similar results were achieved for GS where, in addition, long plasma exposure also led to higher cell numbers. For AF, a modest but significant increase was observed for 60 s for PI. When imaged 48 h post-onset of the cultures, more terminally dead cells were observed, especially for AT and GS (Supplementary Figure S1). Following this, these investigations were performed for the indirect regimen, where the cells were imaged directly on the plastic surface of the 96-well plate (Figure 3d). Again, there was not much of an increase in PI fluorescence for all three dental composite types in the gas plasma conditions, while H_2_O_2_ showed significantly greater cytotoxicity when compared to the direct regimens (Figure 3e).

### 2.3. Secretion Profiling of Three Types of Gas Plasma-Treated Dental Composites

**Figure 4:**
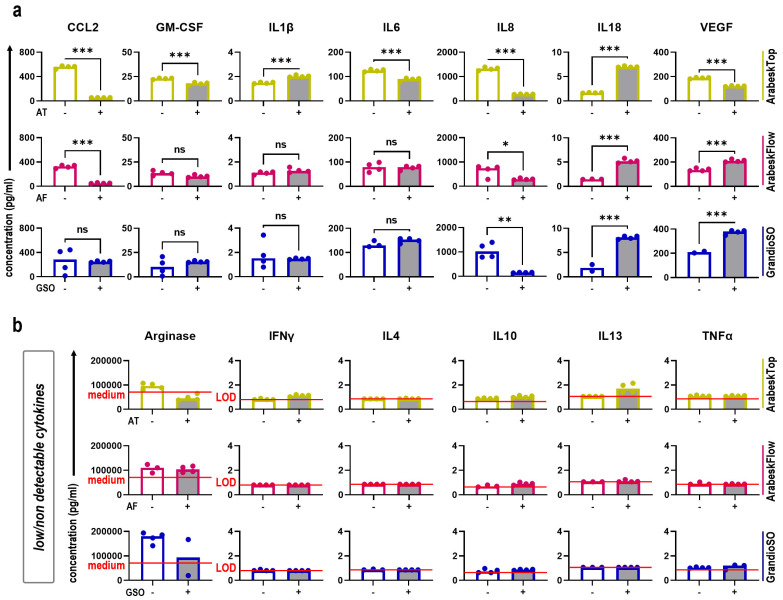
**Secretion profiles of cells cultured in presence or absence of composite chips.** (**a**,**b**) good to highly abundant (**a**) and modest to low abundant (**b**) absolute concentrations of 13 different cytokines and chemokines in cellular supernatants 24 h after onset of human HaCaT keratinocytes cultured either on untreated composite chips or in the absence of composite chips in tissue culture-treated 96-well plates. Data are mean of four technical replicates pooled from six independent experiments with three technical replicates each. Statistical analysis was performed using *t*-test. LOD = (lower) limit of detection. * = *p* < 0.05, ** = *p* < 0.01, and *** = *p* < 0.001.

**Figure 5:**
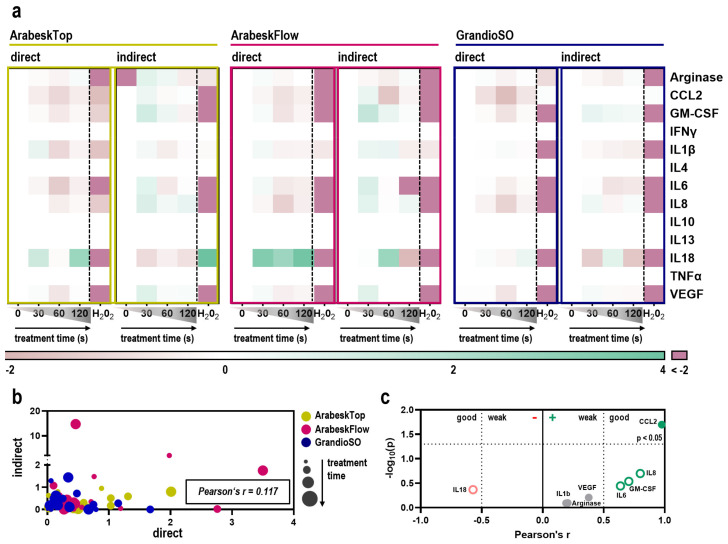
**Cellular secretion profiles of gas plasma-treated composite chips.** (**a**) levels of 13 different cytokines and chemokines in cellular supernatants 24 h after onset of human HaCaT cultures on gas plasma-treated (30 s, 60 s, 120 s), hydrogen peroxide (H_2_O_2_)-spiked composite chips (direct), or human HaCaT cells cultured in the absence of composite chips but in the presence of cell-culture medium previously incubated with untreated or gas plasma-treated composite chips (indirect), normalized to untreated conditions (0); (**b**) Pearson’s correlation analysis of treatment mode (absolute direct vs. indirect fold changes in positive numbers of better visualization) for the three composite types and gas plasma treatment times used in this study; (**c**) Pearson’s correlation analysis of absolute chemokine and cytokine levels of the direct treatment regimen supernatants with metabolic activity data at 24 h of the direct treatment regimen. Data are mean of four technical replicates pooled from six independent experiments with three technical replicates each.

Next, to assess the impact of gas plasma-treated dental composites on human HaCaT keratinocytes with regard to the inflammation-related secretion of cytokines and chemokines, we investigated the supernatants of the cells. When comparing the secretion profiles between cells cultured in the presence or absence of composite chips, the concentration of several soluble mediators was significantly altered (Figure 4a). Consistent changes between all three dental composites were found for IL8 and IL18, while for CCL2 and VEGF, two out of three materials presented similar results. AT showed the most differences. Several cytokines were present only at low concentrations or were below the detection threshold (Figure 4b). An exception was arginase, which is already present in cell culture medium, but significant changes due to the composite chips were not observed. Gas plasma exposure of dental composite material had profound effects on the human HaCaT keratinocyte secretion profiles (Figure 5a). Except for the mediators released only at or below the detection threshold, all other targets showed increased or decreased levels in the gas plasma groups. In general, H_2_O_2_ showed the most dramatic changes, as evident by a firm (>2-fold) decrease in at least four cytokines or chemokines for either of the composite types and direct or indirect regimens. Concerning the gas plasma conditions, an increase was observed in many samples for IL18, and for the indirect regimens for IL6, IL8, and GM-CSF. In the direct regimen, gas plasma dental composite treatment mostly led to decreased levels, especially of CLL2, GM-CSF, IL6, IL8, and VEGF. A non-linear effect between the gas plasma treatment times was observed in many of our results. In this regard, the 60 s exposure time often had a more substantial effect than the 30 s, while the 120 s was more similar to the 30 s. Additionally, the 60 s showed more remarkable changes in the secretion profiling than the 120 s in many samples, e.g., IL6, IL8, VEGF, and CCL2. To understand the relation between direct and indirect regimens, we performed Pearson’s correlation analysis across all composite types and gas plasma treatment times (Figure 5b). No correlation was found, indicating different mechanisms at play within each treatment regimen. Finally, we performed correlation analysis for the gas plasma exposure time-related metabolic reduction rates and total cytokine and chemokine concentrations in the direct treatment regimen (Figure 5c). IL18, IL8, GM-CSF, and IL6 showed a good but non-significant correlation, while CCL2 correlation was highly significant (*p* < 0.05). Secretion profiling at 48 h for the direct regimen showed similar, although not completely congruent results (Supplementary Figure S2).

### 2.4. Composite Particle Release Analysis

**Figure 6:**
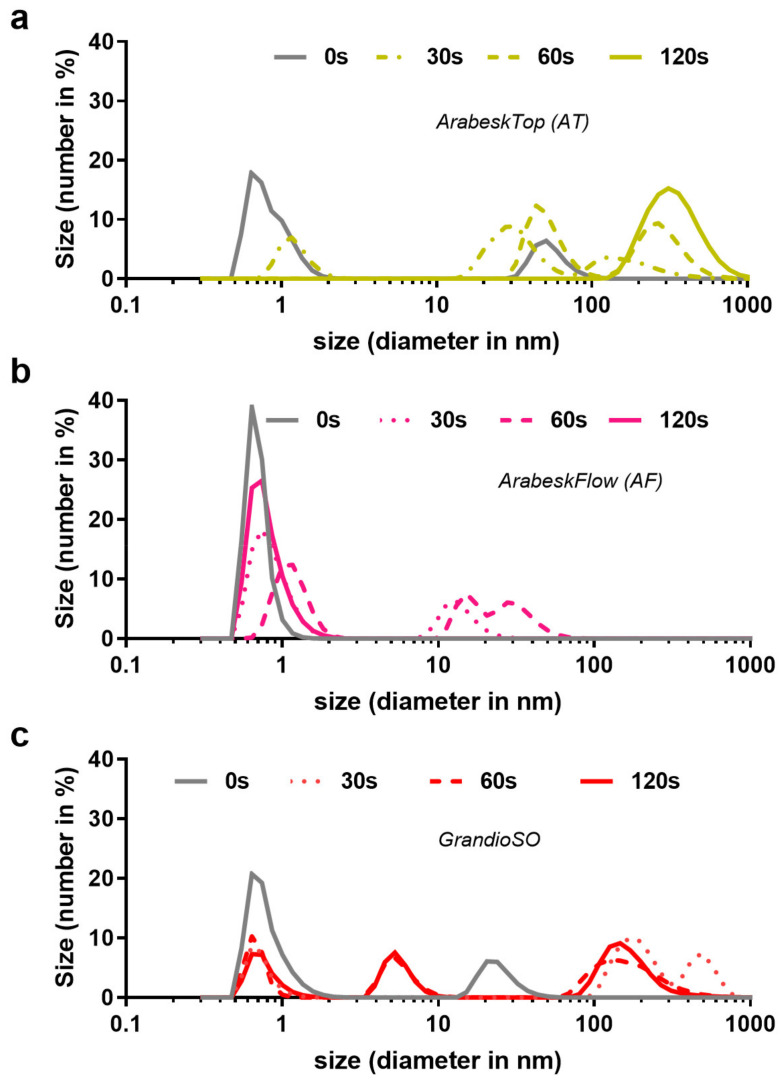
**Resin particle release into buffered solution.** (**a**–**c**) particle size estimations using dynamic light scattering (DLS) of untreated and gas plasma-treated (30 s, 60 s, and 120 s) AT (**a**), AF (**b**), and GSO in phosphate-buffered saline, in which the materials were incubated for 24 h prior to analysis.

To investigate whether gas plasma treatment of composites triggered the release of composite material, dynamic light scattering analysis was performed in phosphate-buffered saline in which the materials were incubated for 24 h. In untreated AT, a small peak of particles at about 50 nm was detected (Figure 6a). Gas plasma treatment yielded a release of particles substantially larger in a treatment time-dependent fashion, with 120 s of exposure generating the biggest fraction at approximately 300 nm. This was not the case for AF (Figure 6b), where untreated composite did not show a second, larger peak, and 30 s and 60 s, but not 120 s, generated particles sized between 10–30 nm. The reason for this remains to be explored. Similar to AT, untreated GSO showed a small fraction of larger particles, in this case at approximately 20 nm (Figure 6c). Gas plasma exposure of 30 s, 60 s, and 120 s led to two new particle peaks at approximately 50 nm and 200 nm. Whether these particles are directly related to the biological effects observed remains to be established in future work.

## 3. Discussion

Gas plasma applications targeting various diseases and conditions are of increasing interest in dentistry. Dental composites are biocompatible materials frequently present in the oral cavity, but the biological consequences of unintended composite gas plasma treatment are unknown. To this end, the current study explored the toxicity and inflammatory mediator release in human HaCaT keratinocytes cultured on three different gas plasma-treated composite chips.

We observed a significant but overall modest metabolic activity reduction in keratinocytes cultured directly on the gas plasma-treated composite surface. Gas plasma is an enabling technology for surface modifications in many industries [15,16,17]. Hence, such modifications might account for the reduction in metabolic activity. Our high content imaging suggested a similar or enhanced number of cells on the surface. This is in line with previous findings of the plasma jet used in our study, where enhanced HaCaT keratinocyte growth was observed on gas plasma-treated polystyrene surfaces [18]. In conjunction with the relatively low increase in the terminal cell death marker PI on cells on the surface of gas plasma-treated composites, it seems plausible that these surfaces caused growth arrest rather than cell death. In previous studies, such cell cycle arrest was found in HaCaT keratinocytes exposed to gas plasma or plasma-treated medium using the current and other plasma systems [19,20]. The indirect regimen was set up and applied to test the hypothesis if gas plasma-sputtered composite particles dissolve into liquids and mediate effects. However, no toxicity was observed in this regime. Hence, we believe most of the effects to be a consequence of direct composite surface-to-cell interaction. Many other studies have been performed in the context of gas plasma treatment and its effects on HaCaT keratinocytes [21,22,23,24], indicating that this cell type may be suitable as an indicator of reactive species and their mediated modifications.

It was interesting to note that culturing the cells in the presence of composite material compared to plain culturing in tissue culture plastic affected the release of several cytokines and chemokines, such as CCL2, IL6, IL8, IL18, and VEGF. In untreated vs. gas plasma-treated composites, the same analytes were found to be released differentially. IL8 (CXCL8) is increased upon oxidative stress and TNFα-induced keratinocytes differentiation as well as toll-like receptor engagement [25,26,27]. It is an alarming factor and chemo-attractant to spur the immigration of professional phagocytes, mainly neutrophils, into damaged or inflamed tissues [28,29]. The fact that its baseline release levels decreased in the presence of composite and decreased even more if composites were gas plasma-treated suggests an anti-inflammatory effect of both. Accordingly, the indirect regimen only had a minor IL-8 stimulating effect, suggesting the possibility that gas plasma-treated composite-derived compounds may have diffused into the culture media to mediate modest biological effects. The IL8 levels highly correlated to the metabolic activity reduction data, underlining IL8′s sensitivity as a marker molecule. The only marker with a higher correlation was CCL2, which showed trends similar to IL8 (i.e., reduction in the presence of composite and gas plasma exposure and mostly unchanged or a modest increase in indirect regimens). CCL2′s synonym, monocyte-chemoattractant protein 1 (MCP-1), reveals that it attracts monocytes to inflammation sites to prime their maturation and differentiation [30]. Similarly, granulocyte-macrophage stimulating factor (GM-CSF) supports cell proliferation and acts as a chemo-attractant for neutrophils and myeloid cells [31,32]. Its release patterns were highly similar to IL8 and CCL2, suggesting that dental composites reduced overall cellular activity and inflammatory signaling via cytokine and chemokine release with an additional decrease for gas plasma treatment. The only prominent exception to this tendency was the consistently elevated IL18 levels, a cytokine associated with caspase-1-mediated pro-IL18 cleavage and inflammasome activation [33]. Increased IL18 release by human keratinocytes was found in response to thioglycolate-induced reductive stress and UV-B exposure in vitro [34,35], while reduced levels were found with H_2_O_2_-induced oxidative stress [36], corroborating our study results. It can be speculated that oxidized composite microparticulate triggered the NLRP3-mediated inflammasome activation that would lead to IL18 release, as was recently suggested for microparticles in oral keratinocytes [37]. The indirect regimen only partially recapitulated the direct regimen’s IL18 release changes, which may be due to a higher dilution of any particles released from the composite into the media. Altogether, gas plasma-treated dental composites produced moderate changes in secretion profiles with both pro- and anti-inflammatory properties.

This study addressed the potential safety concerns of plasma technology application in biomedicine. This is in line with several previous studies on the safety of medical gas plasma applications, especially concerning the plasma jet kINPen used in this study. The jet was considered to be safe for applications in humans, based on several patient studies and long-term follow-ups [38,39,40]. This includes a lack of excessive thermal effects on tissues; an absence of any deficient wound healing or scar formation, safe electric and (UV) light emission profiles; and reproducible results in terms of stability of the generating gas plasma. The recommended kINPen plasma treatment time for chronic wounds and ulcers is 30 s per cm^2^ while moving the jet at a speed of approximately 5 mm per second over the treatment target. This is much less than in our experimental study, where the jet was only pointed (but not moved) towards a target, leading to much longer treatment cycles to a relatively smaller area without any movement. Considering that in oral applications, dental composite gas plasma treatment would be accidental (i.e., nearby the treatment target being, for instance, mucosa, dental implants, or tooth root canals) rather than the main focus, our experimental conditions were intentionally overdosed for the shortest treatment time to explore any biological effects, which were overall minor at 30 s gas plasma exposure time. Regarding the oral cavity, a recent, comprehensive in vivo study with more than 400 mice investigated and gas plasma-treated for 12 months alone or in combination with potential carcinogens concluded that repeated oral exposure to the kINPen plasma is safe and potentially even protects from carcinogenesis [41]. Another one-year follow-up study in mice that had received gas plasma-assisted wound healing support also did not indicate any tumor formation or scaring [42]. Finally, several studies have shown that gas plasma exposure in vitro, independent of the plasma device investigated, does not induce genotoxic effects [43,44,45]. Additionally, human mucosa exposed to gas plasma ex vivo did not show any damage [46]. However, a limitation of our study was a lack of resin surface analysis following gas plasma exposure, which will need to be addressed in future research using polished material.

Collectively, our data suggest significant but overall moderate biological effects of experimentally overdosed gas plasma-treated dental composites on human keratinocytes in vitro. While there were individual differences between the three composite filling materials investigated, their overall effects on cells were mostly similar, pointing to commonly employed fillers as potential mediators of the findings presented. A clinical trial is currently set up with a novel gas plasma jet device for intraoral applications for implant biofilm removal [45], indicating the importance of further studying the safety and efficacy of this novel yet promising medical technology.

## 4. Materials and Methods

### 4.1. Resin-Based Composite Chip Manufacturing

All three types of composite material (ArabeskTop, AT; ArabeskFlow, AF; GrandioSO, GS) tested in this study and with different properties (Table 1) [47] were purchased commercially (Voco, Cuxhaven, Germany). The restoration material was portioned in similar amounts in customized 3D-printed 96-well templates. The entire template was tightly covered with a glass plate, squeezing the composite chips to similar heights across all cavities. Subsequently, composites were hardened manually using a dental UV light device for the time frames indicated for each composite type.

### 4.2. Cell Culture

Human HaCaT keratinocytes (DKFZ, Heidelberg, Germany) were cultured in fully supplemented cell culture medium consisting of Roswell Park Memorial Institute medium (RPMI 1640; Corning, Kaiserslautern, Germany) containing 10% fetal bovine serum (FBS; Sigma-Aldrich, Taufkrichen, Germany), 1% L-glutamine (Corning, Kaiserslautern, Germany), and 1% penicillin and streptomycin (Corning, Kaiserslautern, Germany). Cells were incubated at 37 °C, 95% humidity, and 5% CO_2_. For the experiments, 5 × 10^3^ cells in 125 µL of fully supplemented cell culture medium were added to a composite chip embedded dry (i.e., without any liquid) in a well of a tissue culture-treated flat-bottom 96-well plate (NUNC, Roskilde, Denmark). This was referred to as the direct regimen, as the cells sinking onto the composite chip came in direct contact with the gas plasma-treated surface. In the indirect regimen, untreated or treated composites chips were incubated in cell culture medium for 24 h, and human HaCaT keratinocytes were cultured in these media. As a reference control, each plate contained wells without composite chips where human HaCaT keratinocytes in naïve media (i.e., not in contact with composite chips) attached to the uncovered bottom of the 96-well plate. Cells and supernatants were analyzed 24 h or 48 h later.

### 4.3. Gas Plasma Setup and Exposure

For gas plasma treatment, the atmospheric pressure plasma jet kINPen (neoplas, Greifswald, Germany) was used with argon (purity 99.9999%; Air Liquide, Stralsund, Germany) as a carrier gas. The chemical and physical characteristics are well-described and are previously summarized [48]. For gas plasma exposure of composite chips, a customized and vented 3D-printed template was used, and the chips were placed within the cavities. Reproducible gas plasma treatment was achieved by connecting the argon plasma jet to a high-precision xyz-stage (CNC, Geldern, Germany) that was programmed to hover above the center of each composite chip, traveling down and visually connecting the gas plasma effluent to the composite chip surface for a predetermined time (30 s, 60 s, or 120 s), before rising and automatically traveling to the adjacent well of the template.

### 4.4. Stereo Microscopy

To provide evidence of HaCaT keratinocyte adhesion and growth on the composite chips, 1 × 10^4^ cells were incubated for 48 h in fully supplemented cell culture medium. After that, the medium was removed, and the cells were fixed using 4% paraformaldehyde. Following this crystal violet staining was performed. Subsequently, composite chips were imaged by stereo microscopy (M165 FC; Leica, Wetzlar, Germany) using a 2× Plan Apo corrected objective. Image acquisition was performed using LAX X software (Leica, Wetzlar, Germany).

### 4.5. Metabolic Activity

The metabolic activity of cells was assessed using the resazurin assay 48 h after direct incubation with the plasma-treated composites or incubation with the cell culture media that had been in contact with the plasma-treated composites before. Briefly, 100 µM of 7-hydroxy-3H-phenoxazin-3-on-10-oxid (resazurin; Alfa Aesar, Kandel, Germany) were added to the cells following incubation for 4 h at 37 °C and 5% CO_2_. Viable cells metabolize non-fluorescent resazurin to fluorescent resorufin. Fluorescence was measured at λ_ex_ 535 nm and λ_em_ 590 nm using a multimode plate reader (F200; Tecan, Männedorf, Switzerland).

### 4.6. High Content Imaging

The thickness of the composite chips was sufficiently low as to restore their semi-translucent property. This allowed for quantitative fluorescence imaging with inverted microscopy mounted in a high-content imaging device (Operetta CLS; PerkinElmer, Hamburg, Germany) harboring a micrometer-precision xyz-stage carrying the plate containing the specimens to the optics. DiD (ThermoFisher, Bremen, Germany) fluorescently labeled human HaCaT keratinocytes cultured on the top surface of the composite chips were imaged using a 5x air (NA = 0.15) objective (Zeiss, Jena, Germany) at three different z-planes (50 µm apart) at λ_ex_ 630 nm and λ_em_ 708 ± 52 nm. Propodium iodide (PI; Sigma-Aldrich, Taufkirchen, Germany) was assessed in parallel at λ_ex_ 550 nm and λ_em_ 610 ± 40 nm. Nine fields of view were imaged for each well, covering the entire well area. For data analysis, all z-stacks were merged into a single maximum intensity projection, and all nine fields of view were digitally stitched into a single global image. Using the higher autofluorescence of the composite chip borders, an image region was segmented using algorithm-driven unsupervised image analysis. In this particular image region, for each field of view separately, the DiD-positive human HaCaT keratinocytes were segmented to retrieve the number of cells per image. In the individual segmented cells, the mean fluorescence intensity (MFI) of PI was quantitatively assessed, and the PI MFI was averaged across all cells for each field of view per well. Image and cell segmentation across a total of more than 15,000 microscopy images in this study were performed using algorithms created with Harmony 4.9 software (PerkinElmer, Hamburg, Germany).

### 4.7. Flow Cytometry

To confirm cytotoxic effects on a single cell level in pilot experiments, human HaCaT keratinocytes were harvested in FACS tubes using accutase. Cells were washed three times with cold FACS washing buffer (Miltenyi Biotec, Mönchengladbach, Germany), and cellular viability was determined by adding 1 µM 4′,6-diamidino-2-phenylindole dihydrochloride (DAPI; BioLegend, Amsterdam, The Netherlands). Flow cytometry experiments were performed using a CytoFLEX LX device (Beckman-Coulter, Krefeld, Germany), and data were analyzed using Kaluza 2.1 software (Beckman-Coulter, Krefeld, Germany).

### 4.8. Chemokine and Cytokine Analysis

Supernatants of human HaCaT keratinocytes cultured in the presence or absence of untreated or gas plasma-treated composite chips of three different types were harvested at 24 h, partially at 48 h, and stored at −20 °C until longitudinal analysis. Prior to quantifying 13 different cytokines and chemokines, similar supernatants were pooled from all independent experiments and technical replicates, and four technical replicates were assessed (LegendPLEX; BioLegend, Amsterdam, The Netherlands) per condition and analyte (up to 60 different conditions). The panel consisted of chemokine ligand 2 (CCL), granulocyte-macrophage colony-stimulating factor (GM-CSF), interferon-gamma (IFNγ), tumor necrosis factor-alpha (TNFα), vascular endothelial growth factor (VEGF), and interleukin-1 beta (IL1β), IL4, IL6, IL8, IL10, IL13, and IL18. In addition, arginase, a hydrolase abundantly present in fully supplemented cell culture media, was analyzed. Analysis was performed using flow cytometry (CytoFLEX S; Beckman-Coulter, Krefeld, Germany) and quantification was calculated against a 5-log standard with specific upper and lower limits of detection (LOD) for each analyte.

### 4.9. Statistical Analysis

All experiments were performed three to six independent times, with at least three technical replicates for each treatment condition, composite type, and incubation time. Graphing and statistical analysis were performed using Prism 9.4.0 (GraphPad Software, San Diego, CA, USA). Statistical comparison of two samples was performed using *t*-test. Statistical comparison of two or more samples was performed using one-way analysis of variances (ANOVA) and Dunnett’s post-hoc testing against the untreated control composite sample (0 s). Correlation analysis was performed using Pearson’s correlation coefficient. Levels of significance were indicated as follows: α = 0.05 (*), α = 0.01 (**), α = 0.001 (***).

### 4.10. Photon Correlation Spectroscopy

Photon correlation spectroscopy measurements were performed using a ZS90 dynamic light scattering (DLS) device (Malvern instruments, Kassel, Germany) equipped with a helium–neon laser light source (632 nm). Media incubated with untreated or gas plasma-treated resins were measured in low-volume disposable cuvettes (ZEN0040). DLS measurements were performed at a set angle of 90° and attenuator at 11. The size was measured at 22 °C, with an equilibration time of 120 s and cuvette position at 3 mm. Backscatter angled detection was performed at 173° with a scattering collection angle of 147.7°.

## Figures and Tables

**Table 1:** Dental-resin composite material used in this study.

Name (Abbrev.)	Type	Resin Matrix/Filler Size	Commercial Provider
ArabeskTop (AT)	flowable composite (low filled, <70%)	BisGMA, TEGDMA, UDMA/0.05–1.00 µm	Voco, Germany
ArabeskFlow (AF)	flowable composite (medium filled, 71–79%)	BisGMA, TEGDMA, UDMA/~1.00 µm	Voco, Germany
GrandioSO (GS)	flowable composite (high filled, >80%)	BisGMA, TEGDMA, BisEMA/20–40 µm	Voco, Germany

## Data Availability

Data and printing files for the two 96-well templates for generating composite chips are available from the corresponding author upon reasonable request.

## Supplementary Materials

The following supporting information can be downloaded at: https://www.mdpi.com/article/10.3390/molecules27144519/s1, Supplemental Figure S1. Cellular viability at 48 h. Quantitative image analysis of the mean propidium-iodide (PI) signal intensity of fluorescently labeled HaCaT keratinocytes incubated on the top, gas plasma-exposed composite chip side for 48 h. Data are mean of three independent experiments with three technical replicates and several fields of view each. Statistical analysis was performed using one-way analysis of variances with * = *p* < 0.05, ** = *p* < 0.01, and *** = *p* < 0.001 and Dunnett’s post-hoc test for untreated control conditions (0 s). Supplemental Figure S2. Cellular secretion profiles at 48 h. levels of 13 different cytokines and chemokines in cellular supernatants 48 h after onset of human HaCaT cultures on gas plasma-treated (30 s, 60 s, 120 s) or hydrogen peroxide (H_2_O_2_)-spiked composite chips normalized to untreated conditions (0). Data are mean of four technical replicates pooled from three independent experiments with three technical replicates each.
