# Supplementary material for: Biological Risk Assessment of Three Dental Composite Materials following Gas Plasma Exposure
Source: Molecules. 2022 Jul 15;27(14):4519. doi: 10.3390/molecules27144519 (PMC9322037; doi:10.3390/molecules27144519)
Supplement: Supplementary file 1 [file molecules-27-04519-s001.zip › molecules-1817429-supplementary.pdf]

SI Figures

SI Figure S1

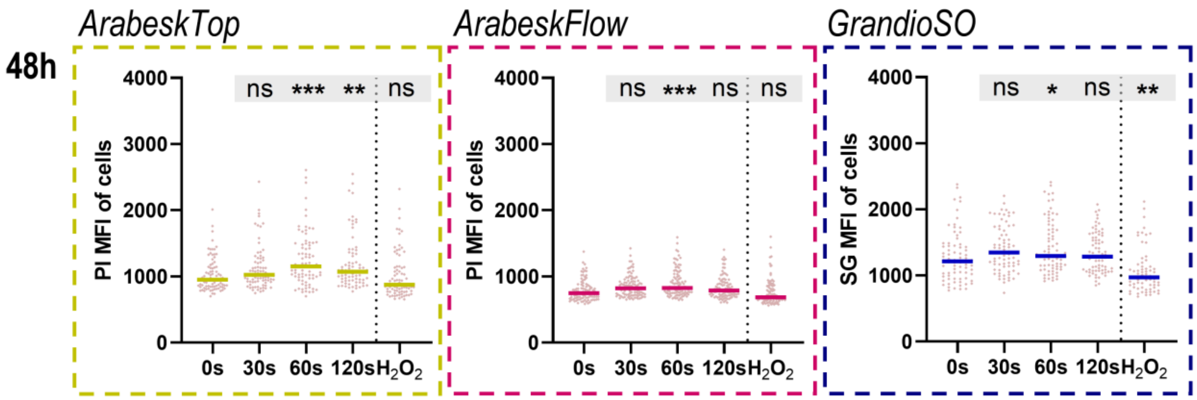

Supplemental Figure S1. Cellular viability at 48h. Quantitative image analysis of the mean propidium-iodide (PI) signal intensity of fluorescently labeled HaCaT keratinocytes incubated on the top, gas plasma-exposed composite chip side for 48h. Data are mean of three independent experiments with three technical replicates and several fields of view each. Statistical analysis was performed using one-way analysis of variances with * = $p < 0.05$, ** = $p < 0.01$, and *** = $p < 0.001$ and Dunnett's post-hoc test for untreated control conditions (0s).

SI Figure S2

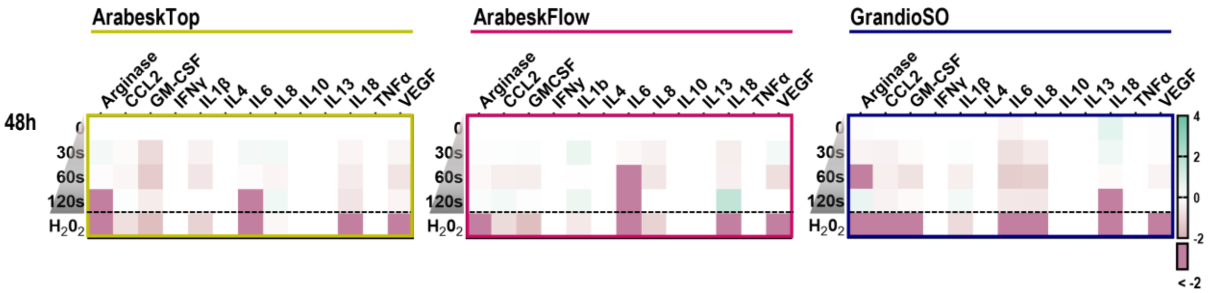

Supplemental Figure S2. Cellular secretion profiles at 48h. levels of 13 different cytokines and chemokines in cellular supernatants 48h after onset of human HaCaT cultures on gas plasma-treated (30s, 60s, 120s) or hydrogen peroxide (H_2O_2)-spiked composite chips normalized to untreated conditions (0). Data are mean of four technical replicates pooled from three independent experiments with three technical replicates each.
